# Evaluation of interfractional shift corrections in Gamma Knife radiosurgery

**DOI:** 10.1002/acm2.70787

**Published:** 2026-09-18

**Authors:** Edward A. Opalko, Dheerendra Prasad, Matthew B. Podgorsak

**Affiliations:** ^1^ Department of Radiation Medicine Roswell Park Comprehensive Cancer Center Buffalo New York USA; ^2^ University at Buffalo The State University of New York Buffalo New York USA

**Keywords:** 3D printing, Gamma Knife Esprit, quality assurance, small field dosimetry

## Abstract

**Purpose:**

The purpose of this study was to evaluate the ability of the GammaPlan treatment planning system used in the Gamma Knife Icon and Esprit machines to automatically correct large interfractional shifts in hypofractionated frameless treatments using a custom 3D printed phantom.

**Methods:**

A custom 3D printed insert was created to modify a commercial head phantom. The water equivalence of polylactic acid (PLA) was determined by comparing optical densities of film using solid water and PLA as buildup. Lesions of varying size, shape and location were created on images of the phantom to replicate several clinical scenarios. Seven 5‐fraction plans were generated using typical dose objectives. Six combinations of headrests/masks were created to simulate different setup positions. Masks were changed between fraction measurements to simulate interfractional shifts. The composite dose distributions of the corrected plans were measured using Gafchromic film and compared to the original dose distribution using Gamma Analysis.

**Results:**

The average deviation for Coverage, Paddick Conformity Index and Gradient Index across all plans remained unchanged between the corrected plan and the original plan. No deviation was greater than 0.01 between the three metrics. Compared to the original plans, the measured dose distributions produced an average Gamma Passing Rate of 98.3% for 3%/1mm, 99.6% for 2%2mm, 96.3% for 2%1mm and 92.7% for 1%/1mm. All but two dose distributions produced passing rates above 90%. For the dosimetric analysis of PLA, the optical densities at various MU values were found to be equivalent for solid water and PLA.

**Conclusion:**

The correction algorithm was able to produce a corrected plan almost identical to the original plan in terms of plan statistics and dose distributions. The algorithm could accurately correct for extreme interfractional shifts beyond what is seen clinically. The results of this study also show the potential for PLA phantoms to be used as dosimetry tools.

## 1 INTRODUCTION

The Leksell Gamma Knife Icon and Gamma Knife Esprit, (Elekta AB, Stockholm, Sweden) allow for hypofractionated treatments using a noninvasive fixation mask as an immobilizer instead of the traditional fixation frame. This is due to the integrated CBCT system which is used to define the Leksell Stereotactic Coordinate System. Additionally, the CBCT system functions as a pre‐treatment imaging tool which allows images to be taken prior to each fraction to determine translations or rotations in the patient's position with respect to their position in the reference CBCT.

During a hypofractionated workflow, patient plans are initially designed using CT or MRI images. The patient is then set up on the treatment couch with a thermoplastic fixation mask and headrest molded to their position. A reference CBCT is taken and used to define the stereotactic coordinate system. The reference CBCT is registered to the planning images which assign stereotactic coordinates to each shot isocenter created in the initial treatment plan. These shot coordinates are based on the patient's position during the reference CBCT. For subsequent fractions, a setup CBCT will be taken and registered with the reference CBCT to determine any interfractional shifts that occur between the patient's daily position and their reference position. The translational and rotational shifts determined by the rigid registration algorithm are used to modify and correct shot coordinates to preserve the planned shot position with the patient's anatomy based on their daily position.^1^ The corrected coordinates are calculated using the formula:

(1) $$v^{\prime }=H*v$$

where $v^{\prime }$ is the corrected shot isocenter position, v is the planned shot isocenter position, and H is the transformation matrix:

(2)

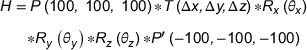




This transformation matrix is based on the rotations and translations from the registration through the isocenter position (100, 100, 100) where P is the isocenter position matrix, T is the translation matrix for translations in the x‐axis (Δx), y‐axis (Δy) and z‐axis (Δz), $R_{x}\left(\theta_{x}\right)$ is the rotation matrix for the angle of rotation about the x‐axis, $R_{y}\left(\theta_{y}\right)$ is the rotation matrix for the angle of rotation about the y‐axis, $R_{z}\left(\theta_{z}\right)$ is the rotation matrix for the angle of rotation about the z‐axis, and $P^{\prime }$ is the inverse isocenter position matrix.

Within the 4 × 4 transformation matrix, the first three dimensions represent the spatial components in the x‐, y‐, and z‐directions, given in millimeters. The fourth component is a dimensionless, homogenous coordinate that allows translations to be incorporated into the matrix multiplication with rotations. This helps preserve the physical units of the resulting product.

The dose is then recalculated using the corrected shot coordinates and displayed for approval. In the current version of the Leksell GammaPlan (version 11.4.1) treatment planning system, the corrected shot coordinates are not displayed on the treatment console during treatment. Instead, the coordinates from the original plan are displayed with no way to verify the corrected coordinates in real time and can only be accessed post‐delivery via log files.

Several studies have aimed at verifying the shot correction algorithm.^2^, ^3^ Hu et al. induced interfractional rotations and translations and compared the adapted result to the original plan, finding good agreement between the corrected fraction and the original fraction.^3^ This study, however, only looked at two lesions and did not examine the impact of the corrected shot coordinates on plan statistics. The purpose of this work is to evaluate the accuracy of GammaPlan's shot correction algorithm for several clinical scenarios using a 3D printed phantom.

## 2 METHODS AND MATERIALS

### 2.1 Phantom modification and creation

**FIGURE 1:**
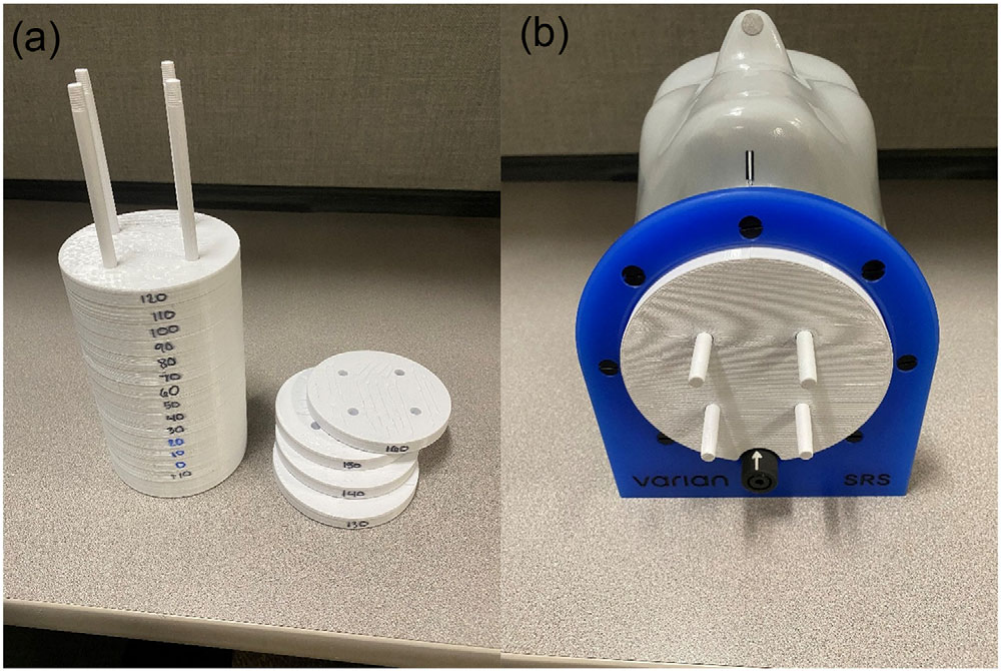
(a) A custom 3D printed insert made with PLA at 92% infill density. The design includes layers of stacked disks for systematic film placement for single and multi‐lesion dosimetry. (b) The 3D printed insert fits securely within a commercial SRS head phantom.

A custom 3D printed insert, as shown in Figure 1, was created to fit within a commercially available SRS head phantom (Varian Medical Systems, Palo Alto, CA), an anthropomorphic phantom designed for end‐to‐end SRS measurements. The insert design comprises of a series of 1 cm thick slabs stacked around four positional rods. This feature allows for radiochromic film to be placed systematically along the length of the phantom, enabling multi‐lesion dosimetry measurements. The insert can then be placed within the cavity of the original phantom and locked in place to prevent movement. A model of the design was generated using Autodesk Fusion (Autodesk, San Francisco, CA). The insert was 3D printed using polylactic acid (PLA) with a Pulse E‐111 3D Printer (Mattherhackers, Lake Forest, CA).

### 2.2 Dosimetric evaluation of PLA

The phantom insert was 3D printed at 92% infill density. The average density of standard PLA filament is roughly 1.24 g/cm^3^. Previous studies have shown that PLA can produce dosimetric characteristics similar to water when printed at lower infill densities.^4^, ^5^, ^6^, ^7^, ^8^, ^9^, ^10^ Mukwada et al. created an anthropomorphic head phantom using PLA with infill densities ranging from 83%‐100% to simulate different anatomical structures. They found PLA printed at 93% infill density produced HU numbers similar to soft tissue.^7^ In‐house research within our department has shown PLA phantoms printed at 92% infill density produce HU numbers similar to water and soft tissue.

At 92% infill density, 8% of the volume will be air. The total volume of the insert is approximately 1650 cm^3^, so the total volume of air is 132 cm^3^, spread throughout the insert. Since the print was done using a nozzle size of 0.4 mm, the slicer will calculate an air gap of 0.032 mm (0.4 mm x 8%) between extrusion lines. These gaps are generally spaced at 0.4 mm, the width of each extrusion line. The height of each air gap is 0.1 mm, equal to the layer height set prior to printing. Because the air gaps are limited to 0.032 mm pores, the print is essentially solid, and any air gaps should not disrupt charged particle equilibrium.

The dosimetric characteristics were evaluated by comparing the optical density differences of irradiated Gafchromic film (Ashland, Bridgewater, NJ) using both solid water and PLA as buildup materials. The films were irradiated using a standard setup of a 10 cm x 10 cm field size with a 6 MV photon beam at several doses ranging from 0 to 1600 MU. The film was initially irradiated under 1.5 cm (d_max_) of solid water. A separate piece of film was then irradiated under 1.5 cm of PLA printed at 92% infill density. After 24 h, the optical densities of both film sets were measured using an X‐Rite 301 Transmission Densitometer (X‐Rite, Inc., Grandville, MI).

### 2.3 Lesion and mask creation

Eleven lesions over seven plans were manually generated on a CT image of the phantom. The lesions were created with varying size, shape, and location to represent and test a wide variety of clinical scenarios. Treatment plans were designed in GammaPlan using the standard clinical criteria for metastatic brain treatments. GTV prescription doses ranged from 18 to 24 Gy with the Coverage metric, the percentage of lesion volume receiving the prescription dose, at least 0.95 for all lesions. The Paddick‐Conformity Index (PCI) and Gradient Index (GI) were also recorded. The GI for P5‐L1 was undefined due to the small volume of the lesion. The maximum dose for each lesion was kept under 40 Gy to stay within the dynamic dose range of Gafchromic EBT‐XD. A summary of plan statistics is given in Table 1.

**TABLE 1:** Summary of dose statistics and plan quality indices such as Coverage, Paddick Conformity Index (PCI) and Gradient Index (GI) for each of the eleven manually created lesions.

Lesion	Min. Dose (Gy)	Max. Dose (Gy)	Mean Dose (Gy)	Coverage	PCI	GI
P1	12.8	46.2	29.0	0.97	0.75	2.52
P2	15.9	37.5	26.2	0.99	0.45	3.20
P3	11.2	31.0	22.9	0.98	0.55	3.31
P4	12.1	37.2	25.4	0.97	0.82	2.50
P5‐L1	18.8	36.1	28.3	0.98	0.50	3.70
P5‐L2	8.6	16.1	12.7	0.99	0.50	3.72
P5‐L3	14.8	25.3	22.4	0.98	0.06	—
P5‐L4	11.5	27.8	21.2	0.99	0.72	2.79
P7	10.9	31.9	22.2	0.98	0.70	2.61
P8‐L1	15.0	27.7	21.3	0.98	0.72	3.12
P8‐L2	14.0	36.7	25.2	0.98	0.79	2.66

**FIGURE 2:**
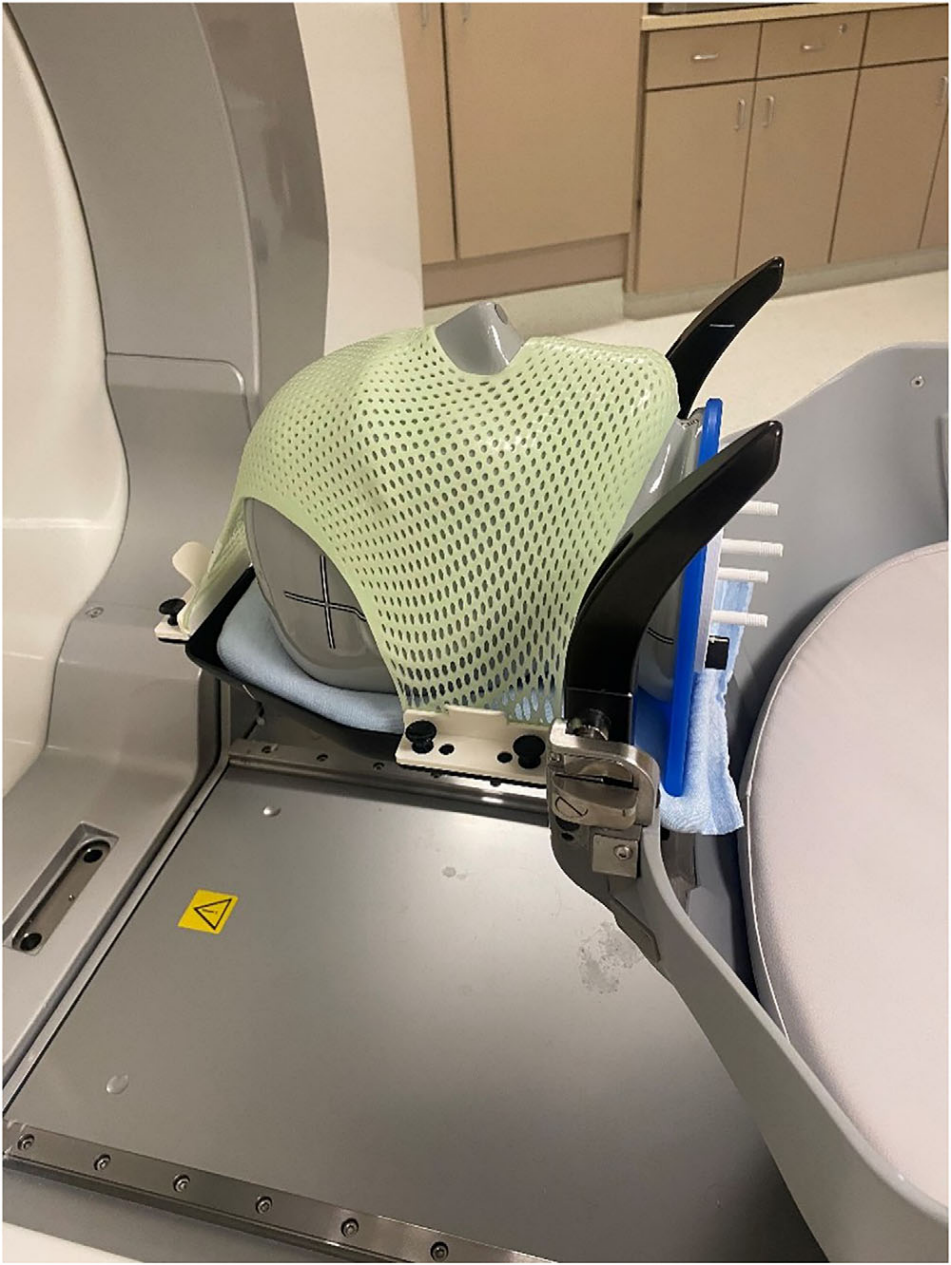
Fixation masks on the phantom were used to replicate patient positions. Six masks were created and interchanged between fractions to simulate interfractional shifts.

Interfractional shifts were created by molding six fixation masks and headrests around the phantom in various positions shown in Figure 2. The rotations and translations induced were significantly larger than clinical scenarios to test the limits of the software's correction algorithm. Mask #1 was designated as the reference position where the stereotactic space was defined within the reference CBCT image. Pieces of EBT‐XD film were cut and placed at the appropriate z‐slice within the phantom corresponding to the location of the lesion(s). The initial plan was delivered with five pieces of film so each fraction could be measured separately. This was done to individually evaluate the positions of the molded fixation masks and determine what effect these positions might have on the corrected dose distribution. The remaining six plans had their fractions delivered consecutively on a single piece of film, creating a composite dose distribution measurement over the five fractions. Between fractions, the position of the phantom was modified by swapping the fixation mask and headrest, simulating an interfractional position change. A setup CBCT was performed and registered to the reference image. The rotations and translations determined by the transformation matrix during registration were displayed, recorded and approved. The fraction was then delivered with the corrected coordinates. This process was repeated for all fractions.

After 24 h, the films were scanned with an Epson Expression 13000XL flatbed color scanner (Epson America, Long Beach, CA). The films were processed using modules within SNC Patient (Sun Nuclear Corporation, Melbourne, FL). Gamma analysis was used to evaluate the agreement between the original planned dose distribution exported from GammaPlan and the measured film dose distribution. The gamma passing rates (GPR) were recorded for several gamma criteria, all with dose thresholds set at 10%.

## 3 RESULTS

### 3.1 Dosimetric evaluation of PLA

Table 2 shows the optical density values for the film irradiated with solid water and PLA at 92% infill density as buildup. For each MU value, the optical density for solid water and PLA were equivalent.

**TABLE 2:** Comparison of optical density measurements of Gafrchromic film between 1.5 cm of solid water and 1.5 cm PLA as buildup materials. Measurements were performed for several doses. The uncertainty in OD was determined by the specifications listed in the device's operating manual.

MU DELIVERED	OPTICAL DENSITY (SOLID WATER) ± 0.02 OD	OPTICAL DENSITY (PLA) ± 0.02 OD
0	0.23	0.23
50	0.31	0.31
100	0.38	0.38
200	0.48	0.48
400	0.63	0.63
800	0.83	0.83
1600	1.09	1.09

### 3.2 Evaluation of shot correction algorithm

Table 3 shows the average position of each fixation mask relative to the reference mask (Mask #1). These values were determined using the rigid registration transformation matrix between each setup CBCT and the reference CBCT. The position values were averaged between two and seven data sets depending on how frequently that mask was used in this study. The largest translations in the x‐, y‐ and z‐directions were 5.82 mm, 17.83 mm, and 35.51 mm, respectively. The largest rotations in the x‐, y‐, and z‐directions were 11.79°, 4.01° and 13.30°, respectively. The standard deviation in mask position for each mask over all uses was submillimeter with the largest translational deviation at 0.52 mm and the largest rotational deviation at 0.81°. These values represent the repeatability of a mask's position over several setups.

**TABLE 3:** Position of each custom fixation mask relative to the reference mask (Mask #1), simulating interfractional patient positional shifts. The rotations and translations were determined from the registration between the imaged mask and the reference mask.

Mask #	Rotation/Translation	X‐Axis	Y‐Axis	Z‐Axis
Mask 2	Rotation (°)	2.65	−4.01	9.88
	Translation (mm)	−5.30	−6.92	24.47
Mask 3	Rotation (°)	11.79	0.90	0.79
	Translation (mm)	−1.96	−17.83	35.51
Mask 4	Rotation (°)	−3.95	0.35	2.82
	Translation (mm)	1.54	−14.09	10.21
Mask 5	Rotation (°)	−1.73	1.53	0.59
	Translation (mm)	−5.82	0.13	−13.98
Mask 6	Rotation (°)	−0.78	−1.53	−13.30
	Translation (mm)	−0.74	−1.90	−0.55

After accepting the registration for each setup CBCT, the dose is recalculated using the corrected coordinates determined from Equation 1. The resulting plan statistics and plan quality indices are displayed for approval. Table 4 shows the deviation in plan statistics between the original plan and the delivered 5‐fraction plan. The average changes in Coverage, Paddick‐Conformity Index (PCI) and Gradient Index (GI) were 0, 0 and 0.01, respectively. No single metric deviated more than 0.03. The average changes in the minimum, maximum and mean doses for each PTV were 0.11 Gy, 0.10 Gy and 0.05 Gy. The largest deviation was ‐0.3 Gy which occurred in both the maximum and minimum PTV dose statistics.

**TABLE 4:** Deviations in dose statistics and plan quality indices between the delivered 5‐fraction after interfractional shifts and the original plan. The average was calculated from the absolute value of each deviation.

Lesion	Min Dose (Gy)	Max Dose (Gy)	Mean Dose (Gy)	Coverage	PCI	GI
P1	0.1	−0.1	0	0	0	−0.01
P2	0.2	0	0.1	0	0	−0.01
P3	−0.1	−0.3	−0.1	−0.01	0.01	0.03
P4	0.2	0.1	0	0	0	0
P5‐L1	−0.3	−0.1	−0.1	0	0.01	0
P5‐L2	0.1	0.1	0.1	0	0	−0.01
P5‐L3	0	0.1	0	0	0	—
P5‐L4	0	−0.1	0	0	0	−0.01
P7	0	−0.1	−0.1	0	0	0
P8‐L1	−0.1	0.1	0	0	0.01	0
P8‐L2	−0.2	0.1	0	0	0	0.01
Average	0.11	0.10	0.05	0.00	0.00	0.01

The delivered plans on Gafchromic film were analyzed and compared to the original dose distribution using gamma analysis. Table 5 shows the GPR for each lesion using several gamma criteria. Since each fraction in Plan 1 was measured separately, the dose for the original 5‐fraction plan was scaled to match the dose for an individual fraction. The average GPR for all 15 analyzed film distributions was 98.3% at 3%/1 mm, 99.6% at 2%/2 mm, 96.3% at 2%/1 mm and 92.7% at 1%/1 mm. Lesions P5‐L3 and P8‐L1 produced GPRs of 81.8% and 78.8%, respectively, using a 1%/1 mm criteria. All other lesion/criteria comparisons produced GPRs above 90%.

**TABLE 5:** Gamma Passing Rates for each measured dose distribution compared to the original dose distribution using several gamma criteria. Each fraction for P1 was measured separately while the remaining lesions were measured as a 5‐fraction composite dose distribution.

Plan/Lesion	3%1mm	2%2mm	2%1mm	1%1mm
P1‐L1	97.8	100	95.5	94.8
P1‐L2	98.1	99.5	91.0	90.5
P1‐L3	99.8	99.8	97.7	97.0
P1‐L4	97.2	99.8	91.7	91.3
P1‐L5	99.1	99.3	96.5	95.3
P2	98.7	98.7	98.7	98.2
P3	98.3	100	97.5	94.0
P4	100	100	100	100
P5‐L1	98.0	98.9	94.5	93.2
P5‐L2	94.5	99.7	94.0	91.7
P5‐L3	99.5	99.9	99.3	81.8
P5‐L4	97.8	99.7	96.0	94.0
P7	97.5	99.1	95.7	93.9
P8‐L1	98.1	99.7	96.7	78.8
P8‐L2	99.9	99.9	99.4	96.1
**Average**	**98.3**	**99.6**	**96.3**	**92.7**

## 4 DISCUSSION

GammaPlan uses TMR10 as the default dose calculation algorithm, which assumes all material to be water. To accurately verify the treatment planning system (TPS) correction algorithm using absolute dose measurements, the phantom and custom 3D printed insert must simulate water as closely as possible. When comparing solid water and PLA as attenuating materials using film, their OD measurements were found to be identical at a 1.5 cm depth as shown in Table 2. The film comparisons also showed a close agreement between PLA and water at larger depths as dose distributions along the central axis of the phantom had undergone at least 5 cm of attenuation since the PLA insert has a radius of 5 cm. Additionally, CT images showed PLA at 92% infill density has a CT number of approximately 30 HU, which is in the expected range for brain tissue. These findings are consistent with previous studies, showing that PLA has water equivalent properties when manufactured in specific ways.^4^, ^5^, ^6^, ^7^, ^8^, ^9^, ^10^ This makes PLA and 3D printing suitable options for dosimetric QA tools.

Each of the five non‐reference fixation masks were designed to simulate interfractional translations and rotations larger than most clinical scenarios. The range of translational shifts induced for this study were 0.55 mm – 35.51 mm while the range of angular shifts was 0.59°‐13.30°, creating extreme scenarios for the correction algorithm. A retrospective study from Bush et al. found that interfractional shifts are submillimeter.^11^ This is consistent with our findings that showed the largest deviation in position between successive setups using the same mask was 0.52 mm and 0.81°. Bush et al. also found that translational shifts mostly occur in the z‐direction with the largest interfractional shift from 124 patient treatments being 7.94 mm. The largest angular shift from the study was found to be 4.82°.^11^

The automatic position correction algorithm summarized by Equation 1 was verified by evaluating the changes in plan statistics along with film measurements comparing the delivered 5‐fraction plan to the original plan. The changes in plan statistics and plan quality indices were evaluated prior to each fraction using the new fixation position. Table 4 shows that all dose statistics and plan quality indices remained essentially unchanged across each fraction with the largest change in any dose metric being 0.11 Gy. The film measurements also showed agreement between the corrected plan and the original plan as the dose distributions analyzed produced acceptable GPRs for each of the four gamma criteria used. These results are consistent with Hu et al. which found dose distributions displaced by clinically large interfractional shifts produced GPRs above 97% at 1%/1 mm^3^ AAPM TG218 and AAPM‐RSS Medical Physics Practice Guideline 9.b recommend that PSQA passing rates be > 90% using a 3%/1 mm criteria for SRS cases.^12^, ^13^ In this study, the average passing rate for 3%/1 mm was 98.3% with no film measurement producing a passing rate below 94%.

Lesions P5‐L3 and P8‐L1 were the only two profiles to produce GPRs below 90% using the strictest gamma criteria of 1%/1 mm. When analyzing the failing points across both dose distributions, the largest contributing factor towards the higher gamma index value was absolute dose. Small pieces of radiochromic film are highly susceptible to curling around the edges which can produce film scanning errors that lead to absolute dosimetric differences up to 4%.^14^ As lesions P5‐L3 and P8‐L1 were both lateral lesions near the edge of the scanned film, this is most likely the cause of the lower passing rates. For the other lesions, there was no discernable correlation between passing rates and lesion size, shape and location proving the correction algorithm is valid for most clinical cases.

The findings from this study verify the accuracy of GammaPlan's positional correction algorithm, even for extreme cases of interfractional setup differences. In cases where the fixation mask is deteriorated, deformed or structurally compromised resulting in large setup deviations, clinical staff can trust the TPS to correct for an updated mask position without the need to replan and reoptimize. This ultimately saves valuable time and resources while the patient is on the treatment couch. Despite this, careful evaluations of corrected plan statistics, dose volume histograms and isodose displays are vital prior to fraction approval, especially when related to organs at risk. The geometric configuration of the system results in discrepancies in the low isodose regions between the corrected and planned distributions while the overall volume of each region should remain the same.^1^ The discrepancies at higher isodose regions should remain the same, which was confirmed in this study.

## 5 CONCLUSION

A custom 3D printed insert was created to modify an existing commercial phantom. Evaluations of the dosimetric properties of PLA showed its capabilities as an alternative to water‐equivalent materials when printed at 92% infill density, expanding the possibilities of 3D printing as a dosimetry tool. The modified phantom was then used to evaluate GammaPlan's positional correction algorithm by creating large setup differences between fractions for sample plans. The corrected plans were found to be in good agreement with the original plans for both plan statistics and the resulting dose distributions.

## AUTHOR CONTRIBUTIONS

**Edward A. Opalko**: Concept and design of study; data acquisition and analysis; manuscript drafting. **Dheerendra Prasad**: Concept and design of study; data analysis; manuscript revision. **Matthew B. Podgorsak**: Concept and design of study; data analysis; manuscript revision.

## CONFLICT OF INTEREST STATEMENT

The authors declare no conflicts of interest.

## Data Availability

The data that support the findings of this study are available from the corresponding author upon reasonable request.
